# Perforator-Related Risk Factors for Perfusion-Related Complications in DIEP Flap Breast Reconstruction^☆^

**DOI:** 10.1016/j.jpra.2025.03.015

**Published:** 2025-03-24

**Authors:** Dernas Suhail (DS), Ryan Faderani (RF), Afshin Mosahebi (AM), Augustine Akali (AA)

**Affiliations:** 1 Morriston Hospital, Swansea, UK, SA6 6NL; 2 Department of Surgery and Inteventional Sciences, University College London, London, UK, NW3 2PS; 3 Department of Plastic Surgery, Royal Free Hospitals NHS Trust, London, UK, NW3 2QG; 4 Department of Plastic Surgery, Hull University Teaching Hospitals NHS Trust, Hull, UK, HU16 5JQ

**Keywords:** Perforator flap, Blood supply, Mammaplasty, Epigastric arteries, Female

## Abstract

**Introduction:**

Despite the advancements in deep inferior epigastric artery perforator (DIEP) flap breast reconstruction, perfusion-related complications (PRCs) remain a concern. Such complications can negatively impact the aesthetic outcome, necessitate revisional surgery and delay adjuvant chemotherapy and radiotherapy. The aim of this study was to investigate the impact of perforator diameter, perforator row, and the shortest distance between the perforator and flap edge on the development of PRCs in DIEP flap breast reconstruction.

**Methods:**

This cohort study prospectively analysed the stored data for consecutive patients who underwent unilateral DIEP flap breast reconstruction from January 2008 to January 2023. Variables with *p*<0.25 in univariate analysis were included in multivariate logistic regression analysis to identify the risk factors for PRCs. *p*<0.05 was deemed statistically significant. Receiver operating characteristic (ROC) curve analysis was conducted to identify the critical distance between the perforator and flap edge distinguishing the PRCs.

**Results:**

V Overall, 292 cases of unilateral DIEP flap breast reconstruction were identified, with a mean patient age of 52.6 years. PRCs occurred in 72 cases (24.7%). Multivariate logistic regression identified the shortest distance between the perforator and flap edge that has a significant impact on PRC incidence. ROC curve analysis found the critical shortest distance between the perforator and flap edge distinguishing PRCs to be 42.5 mm.

**Conclusions:**

This study showed that the shortest distance between the perforator and flap edge is a strong predictor for PRCs in DIEP flap breast reconstruction. As several perforator characteristics are considered when designing and performing DIEP flap breast reconstruction, these findings can guide surgeons in this decision-making process.

## Introduction

The deep inferior epigastric artery perforator (DIEP) flap is currently the gold standard for breast reconstruction. It excels in creating reconstructed breasts with texture, ptosis, mobility, and warmth akin to natural breasts, while keeping donor-site morbidity low.^1^ In autologous reconstruction, a common concern is perfusion-related complications (PRCs), such as fat necrosis, partial flap necrosis and total flap necrosis. PRCs in DIEP flap breast reconstruction can adversely affect the reconstructive and aesthetic outcomes, require additional imaging, lead to revisional surgery, and potentially delay post-mastectomy chemotherapy or radiotherapy.^2^^,^^3^

Developments in surgical technique have facilitated a reduction in PRC rates following DIEP flap breast reconstruction. Between 2000–2010, fat necrosis and flap loss rates were approximately 14% and 3%, respectively.^4^, ^5^, ^6^, ^7^, ^8^ In contrast, from 2011 to 2020, fat necrosis and flap loss rates decreased to approximately 12% and 2%, respectively.^1^^,^^9^, ^10^, ^11^,^12^ Owing to the persistence of PRCs, other facets of the procedure that may impact perfusion and are amenable to modification must be explored.

Comprehending perfusion of DIEP flaps is vital in preventing PRCs. Scheflan and Dinner initially proposed the theory of zonal perfusion for lower abdominal flaps, which was later associated with Hartrampf's name.^13^^,^^14^ The aim of this theory was to aid surgeons in predicting adequately perfused tissue in pedicled transverse rectus abdominis musculocutaneous (TRAM) flaps. Holm et al. later reclassified these zones for DIEP flaps based on clinical perfusion studies.^15^ However, concerns have been raised about the accuracy and applicability of such rigid zoning techniques for DIEP flaps. Following this, Saint-Cyr et al. introduced the perforasome theory, advancing surgeons’ comprehension of flap microcirculation. This theory posits that each perforator-based flap has a distinct vascular territory, termed the perforasome.^16^

When designing a DIEP flap, perforator selection is a crucial factor, considering aspects such as perforator diameter, row, number and relative location within the flap. Despite the importance of these characteristics, there is currently no consensus regarding which perforator characteristics significantly influence DIEP flap perfusion. This study aimed to explore the impact of specific perforator characteristics on the development of PRCs in DIEP flap breast reconstruction.

## Methods

### Study Design

This was a cohort study that used a prospectively maintained database of patients who underwent unilateral DIEP flap breast reconstruction from January 2008 to January 2023 under the care of our senior author (A.A.). This study received local approval as a service improvement project prior to study commencement. The manuscript was prepared in accordance with the Strengthening the Reporting of Observational Studies in Epidemiology (STROBE) guidelines.^17^

This study included consecutive patients who underwent unilateral, unipedicled, immediate DIEP flap breast reconstruction and had a minimum of 6 months follow-up. The exclusion criteria were all cases based on more than one pedicle, significant incomplete data, and patients with <6 months follow-up.

The following patient demographics were collected: age, BMI, co-morbidities, diabetes mellitus status, hypertension, radiotherapy, smoking history and American Society of Anaesthesiologists (ASA) score.

The following operative characteristics were collected: flap weight, operation time, flap ischaemic time and return to operating theatre.

Data on the following perforator characteristics were collected: perforator row, perforator diameter and the shortest distance between the perforator and flap edge. The shortest distance between the perforator and flap edge was used as a metric to describe how central the perforator was located in the flap, with a greater distance between the perforator and flap edge indicating a more centrally located perforator. Perforator diameter was measured intraoperatively using a surgical ruler, and post-operatively confirmed with magnetic resonance angiography (MRA), unless this was contraindicated, in which case computed tomography angiography (CTA) was used. The shortest distance between the perforator and flap edge was measured intraoperatively using a surgical ruler.

### Outcome Measures

The composite primary outcome measure was the rate of PRCs, which included fat necrosis, partial flap necrosis or total flap necrosis. Secondary outcome measures were individual rates of fat necrosis, partial flap necrosis, total flap necrosis and return to theatre.

Flaps were evaluated for the presence of fat necrosis through physical examination by our senior author (A.A.) at the routine follow-up.^18^^,^^19^ Fat necrosis was clinically defined as a palpable nodule or focal area of firmness in the reconstructed breast.^20^ Partial flap necrosis was defined as necrosis of the skin and underlying fat that is <50% of the total flap area.^21^ Total flap necrosis was defined as flap failure requiring surgical debridement of the entire flap.

### Statistical Analysis

Data in the results are reported as means ± standard deviation or as a percentage (%) and ‘n’ represents the number of patients. Independent *t*-tests for continuous variables and Chi-squared tests for categorical variables were performed to compare the means of 2 unrelated groups. Additionally, a multivariate logistic regression model was calculated to account for confounding. Variables with *p*<0.25 in univariate analysis were included in the multivariate logistic regression analysis. *p*<0.05 was deemed statistically significant. Receiver operating characteristic (ROC) curve analysis was performed to identify the cut-off value for the shortest distance between the perforator and flap edge distinguishing the probability of developing PRCs. Area under the curve was also calculated to substantiate the reliability of the cut-off value. A two-sided *p*<0.05 was considered statistically significant. Statistical analysis was performed using IBM SPSS Statistics 28 (New York, US).

## Results

### Patient Demographics

Two hundred and ninety-two patients underwent unilateral DIEP flap breast reconstruction and met the inclusion criteria (Table 1). The mean age was 52.6 years ± 8.7 (range 28.3–75.2 years). BMI ranged from 18.6–45.3 kg/m^2^, with a mean of 28.8 ± 4.8 kg/m^2^. Among them, 3.4% (n=10) of the patients were smokers, whereas 9.9% (n=29) were ex-smokers. Additionally, 2.1% (n=6) of the patients had type 2 diabetes mellitus and 11.6% (n=34) had hypertension. Furthermore, 44.2% (n=129) of the patients were classified as ASA 1, 53.1% (n=155) were ASA 2, 2.7% (n=8) were ASA 3, and no patients were assigned as ASA 4. MRA was the imaging modality of choice for 266 (91%) patients. Patients were followed up for a minimum of 6 months, with an average follow-up period of 8.5 years (range 6 months–15 years).

**Table 1:** Correlation Between Patient Characteristics and Surgical Outcomes.

Outcome: PRCs	No PRCs	PRCs	*p*-Value
Age (years)	53.06 ± 8.9	52.5 ± 8.6	0.642
BMI (kg/m^2^)	28.7 ± 4.7	29.1 ± 5.1	0.553
ASA			0.211
ASA 1	100 (77.5%)	29 (22.5%)	
ASA 2	116 (74.8%)	39 (25.2%)	
ASA 3	4 (50%)	4 (50%)	
ASA 4	0 (0%)	0 (0%)	
Diabetes			0.646
No Diabetes	215 (75.2%)	71 (24.8%)	
Has Diabetes	5 (83.3%)	1 (16.7%)	
Smoking			0.486
Non-Smoker	192 (75.9%)	61 (24.1%)	
Ex-Smoker	20 (69.0%)	9 (31.0%)	
Smoker	8 (80%)	2 (20%)	
Hypertension			0.494
No Hypertension	196 (76.0%)	62 (24.0%)	
Has Hypertension	24 (70.6%)	10 (29.4%)	
Radiotherapy (RTX)			0.195
No RTX	64 (74.4%)	22 (25.6%)	
Pre-Reconstruction RTX	93 (77.7%)	27 (22.5%)	
Post-Reconstruction RTX	33 (66.0%)	17 (34.0%)	
Flap Weight (kg)	0.72 ± 0.45	0.71 ± 0.22	0.799
Perforator Diameter (mm)	3.6 ± 1.0	3.1 ± 0.5	0.028*
Shortest Distance Between Perforator and Flap Edge (mm)	49.6 ± 6.4	39.4 ± 7.3	<0.001*
Perforator Row			0.922
Medial Row	68 (74.7%)	23 (25.3%)	
Lateral Row	43 (75.4%)	14 (24.6%)	
Operation Time (min)	436.8 ± 166.5	409.3 ± 190.1	0.288
Ischaemia Time (min)	64.2 ± 4.2	71.9 ± 7.7	0.032*
Outcome: Fat Necrosis	No Fat Necrosis	Fat Necrosis	*p*-Value
Age (years)	52.6 ± 8.6	52.9 ± 9.1	0.781
BMI (kg/m^2^)	28.8 ± 4.7	28.6 ± 5.0	0.795
ASA			0.448
ASA 1	104 (80.6%)	25 (19.4%)	
ASA 2	125 (80.6%)	30 (19.4%)	
ASA 3	5 (62.5%)	3 (37.5%)	
ASA 4	0 (0%)	0 (0%)	
Diabetes			0.843
No Diabetes	229 (80.1%)	57 (19.9%)	
Has Diabetes	5 (83.3%)	1 (16.7%)	
Smoking			0.420
Non-Smoker	204 (80.6%)	49 (19.4%)	
Ex-Smoker	21 (72.4%)	8 (27.6%)	
Smoker	9 (90%)	1 (10%)	
Hypertension			0.730
No Hypertension	206 (79.8%)	52 (20.2%)	
Has Hypertension	28 (82.4%)	6 (17.6%)	
Radiotherapy (RTX)			0.766
No RTX	66 (76.7%)	20 (23.3%)	
Pre-Reconstruction RTX	97 (80.8%)	23 (19.2%)	
Post-Reconstruction RTX	39 (78%)	11 (22%)	
Flap Weight (kg)	0.72 ± 0.44	0.71 ± 0.23	0.902
Perforator Diameter (mm)	3.6 ± 0.7	3.1 ± 0.4	0.021*
Shortest Distance Between Perforator and Flap Edge (mm)	48.9 ± 6.8	38.4 ± 7.5	<0.001*
Perforator Row			0.660
Medial Row	73 (80.2%)	18 (19.8%)	
Lateral Row	44 (77.2%)	13 (22.8%)	
Operation Time (min)	404.3 ± 202.4	429.4 ± 168.1	0.330
Ischaemia Time (min)	65.4 ± 11.2	72.6 ± 8.8	0.042*

Outcome: Partial Flap Necrosis	No Partial Flap Necrosis	Partial Flap Necrosis	*p*-Value
Age (years)	52.6 ± 8.8	53.6 ± 6.6	0.674
BMI (kg/m^2^)	29.6 ± 4.7	31.3 ± 4.3	0.092
ASA			0.010*
ASA 1	126 (97.7%)	3 (2.3%)	
ASA 2	146 (94.2%)	9 (5.8%)	
ASA 3	6 (75%)	2 (25%)	
ASA 4	0 (0%)	0 (0%)	
Diabetes			0.169
No Diabetes	273 (95.5%)	13 (4.5%)	
Has Diabetes	5 (83.3%)	1 (16.7%)	
Smoking			0.611
Non-Smoker	242 (95.7%)	11 (4.3%)	
Ex-Smoker	27 (93.1%)	2 (6.9%)	
Smoker	9 (90%)	1 (10%)	
Hypertension			0.142
No Hypertension	29 (91.2%)	3 (8.8%)	
Has Hypertension	249 (95.7%)	11 (4.3%)	
Radiotherapy (RTX)			0.004*
No RTX	85 (98.8%)	1 (1.2%)	
Pre-Reconstruction RTX	115 (95.8%)	5 (4.2%)	
Post-Reconstruction RTX	43 (86%)	7 (14%)	
Flap Weight (kg)	0.76 ± 0.40	0.87 ± 0.22	0.213
Perforator Diameter (mm)	3.7 ± 0.7	3.0 ± 0.3	0.032*
Shortest Distance Between Perforator and Flap Edge (mm)	45.7 ± 8.4	38.3 ± 6.0	0.007*
Perforator Row			0.945
Medial Row	88 (96.7%)	3 (3.3%)	
Lateral Row	55 (96.7%)	2 (3.5%)	
Operation Time (min)	423.1 ± 178.5	441.2 ± 90.8	0.574
Ischaemia Time (min)	66.4 ± 6.6	68.0 ± 4.5	0.639

Outcome: Total Flap Necrosis	No Total Flap Necrosis	Total Flap Necrosis	*p*-Value

Age (years)	52.7 ± 8.7	50.9 ± 8.5	0.587
BMI (kg/m^2^)	28.7 ± 4.7	30.6 ± 6.6	0.314
ASA			0.593
ASA 1	127 (98.4%)	2 (1.6%)	
ASA 2	150 (96.8%)	5 (3.2%)	
ASA 3	8 (100%)	0 (0%)	
ASA 4	0 (0%)	0 (0%)	
Diabetes			0.698
No Diabetes	6 (100%)	0 (0%)	
Has Diabetes	279 (97.6%)	7 (2.4%)	
Smoking			0.575
Non-Smoker	246 (97.2%)	7 (2.8%)	
Ex-Smoker	29 (100%)	0 (0%)	
Smoker	10 (100%)	0 (0%)	
Hypertension			0.825
No Hypertension	252 (97.7%)	6 (2.3%)	
Has Hypertension	33 (97.1%)	1 (2.9%)	
Radiotherapy (RTX)			0.657
No RTX	84 (97.7%)	2 (2.3%)	
Pre-Reconstruction RTX	118 (98.3%)	2 (1.7%)	
Post-Reconstruction RTX	48 (96%)	2 (4%)	
Flap Weight (kg)	0.72 ± 0.40	0.63 ± 0.18	0.625
Perforator Diameter (mm)	3.4 ± 0.6	3.2 ± 0.2	0.740
Shortest Distance Between Perforator and Flap Edge (mm)	45.2 ± 8.4	36.0	0.281
Perforator Row			0.166
Medial Row	88 (96.7%)	3 (3.3%)	
Lateral Row	57 (0%)	0 (0%)	

Outcome: Total Flap Necrosis	No Total Flap Necrosis	Total Flap Necrosis	*p*-Value
Operation Time (min)	423.4 ± 177.0	462.8 ± 96.7	0.558
Ischaemia Time (min)	66.4 ± 4.6	76.5 ± 3.9	0.705
Outcome: Return to Theatre	No Return to Theatre	Return to Theatre	*p*-Value
Age (years)	52.7 ± 8.7	51.9 ± 8.5	0.699
BMI (kg/m^2^)	28.7 ± 4.69	29.5 ± 6.22	0.515
ASA			0.551
ASA 1	122 (94.6%)	7 (5.4%)	
ASA 2	143 (78.2%)	12 (21.8%)	
ASA 3	8 (100%)	0 (0%)	
ASA 4	0 (0%)	0 (0%)	
Diabetes			0.514
No Diabetes	267 (93.4%)	19 (6.6%)	
Has Diabetes	6 (100%)	0 (0%)	
Smoking			0.523
Non-Smoker	235 (92.9%)	18 (7.1%)	
Ex-Smoker	28 (96.6%)	1 (3.4%)	
Smoker	10 (100%)	0 (0%)	
Hypertension			0.560
No Hypertension	242 (93.8%)	16 (6.2%)	
Has Hypertension	31 (91.2%)	3 (8.8%)	
Radiotherapy (RTX)			0.447
No RTX	82 (95.3%)	4 (4.7%)	
Pre-Reconstruction RTX	113 (94.2%)	7 (5.8%)	
Post-Reconstruction RTX	45 (90%)	5 (10%)	
Flap Weight (kg)	0.72 ± 0.40	0.68 ± 0.26	0.768
Perforator Diameter (mm)	3.5 ± 0.7	3.3 ± 0.6	0.739
Shortest Distance Between Perforator and Flap Edge (mm)	45.0 ± 8.44	47.1 ± 9.16	0.492
Perforator Row			0.177
Medial Row	85 (93.4%)	6 (6.6%)	
Lateral Row	56 (98.25%)	1 (1.75%)	
Operation Time (min)	422.7 ± 179.4	447.7 ± 105.3	0.549
Ischaemia Time (min)	65.5 ± 64.6	82.5 ± 64.0	0.281

Characteristics of the participants in each group were analysed using the Chi-squared test for categorical variables and independent t-test for continuous variables. Values are displayed as mean ± standard deviation and n (%). Significance (p<0.05) was indicated with an asterisk (*)

The mean mastectomy weight was 709.7 ± 351.9 g (range 131–1691 g). In addition, 53.1% (n=155) of the mastectomies were skin-sparing.

Seventy-two out of the 292 patients (24.7%) had perfusion-related complications. Among the 72 patients, 58 (19.9%) had fat necrosis, 14 (4.8%) experienced partial flap necrosis and 7 (2.4%) had total flap necrosis.

### Perfusion-Related Complications

Associations between demographic factors, operative characteristics, perforator characteristics and PRCs are summarised in Table 1.

Our analysis showed the mean perforator diameter in the PRCs group to be 3.1 ± 0.5 mm, which was smaller than the perforator diameter in the non-PRCs group (3.6 ± 1.0 mm), and this was statistically significant (p=0.028). Additionally, the shortest distance between the perforator and flap edge was smaller among patients who experienced PRCs (39.4 ± 7.3 mm) compared with patients who did not experience PRCs (49.6 ± 6.4 mm), which was also statistically significant (p<0.001). Meanwhile, ischaemia time was longer among patients who experienced PRCs (71.9 ± 7.7 min) versus patients who did not experience PRCs (64.2 ± 4.2 min), and this was also statically significant (p=0.032).

### Fat Necrosis

Associations between demographic factors, operative characteristics, perforator characteristics and fat necrosis are summarised in Table 1.

Our investigation found that the perforator diameter was significantly smaller (p=0.021) among patients who experienced fat necrosis (3.1 ± 0.4 mm) compared with the patients who did not experience fat necrosis (3.6 ± 0.7 mm). Furthermore, the shortest distance between the perforator and flap edge was smaller among the patients who suffered from fat necrosis (38.4 ± 7.5 mm) compared with patients who did not (48.9 ± 6.8 mm), and this was also a statistically significant difference (p<0.001). Additionally, ischaemia time among the fat necrosis group (72.6 ± 8.8 min) was greater than that of the non-fat necrosis group (65.4 ± 11.2 min), and this was also statistically significant (p=0.042).

### Partial Flap Necrosis

Associations between demographic factors, operative characteristics, perforator characteristics and partial flap necrosis are summarised in Table 1.

Our analysis found increasing ASA score to be significantly associated with the development of partial flap necrosis (p=0.010), with 2.3% of ASA 1, 5.8% of ASA 2 and 25% of ASA 3 patients experiencing partial flap necrosis. Radiotherapy status was also significantly associated with the development of partial flap necrosis (p=0.004). Among non-radiotherapy status patients, 1.2%, 4.2% of the patients who received radiotherapy prior to reconstruction and 14% of the patients who received post-reconstruction radiotherapy experienced partial flap necrosis. Perforator diameter was also smaller among the patients who suffered from partial flap necrosis (3.0 ± 0.3 mm) compared with patients who did not develop this complication (3.7 ± 0.7 mm), and this difference was statistically significant (p=0.032). Furthermore, the shortest distance between the perforator and flap edge was significantly smaller (p=0.007) among the patients with partial flap necrosis (38.3 ± 6.0 mm) compared with non-partial flap necrosis patients (45.7 ± 8.4 mm).

### Total Flap Necrosis

Associations between demographic factors, operative characteristics, perforator characteristics and total flap necrosis are summarised in Table 1. We did not identify any significant relationships between these factors and the development of total flap necrosis.

### Return to Operating Theatre

Associations between demographic factors, operative characteristics, perforator characteristics and total flap necrosis are summarised in Table 1. We did not identify any significant relationships between these factors and return to operating theatre.

### Multivariate Logistic Regression Model of Predictive Factors for Perfusion-Related Complications

Through multivariate logistic regression analysis (Table 2), the shortest distance between the perforator and flap edge was shown to be the only independent factor increasing the occurrence of PRCs (OR 0.969, 95% CI 0.940–0.981, p<0.001).

**Table 2:** Multivariate Logistic Regression Model of Predictive Factors for PRCs.

Outcome: PRCs	Odds Ratio (95% CI)	*p*-Value
ASA		
ASA 1	Reference	
ASA 2	0.312 (0.022 - 4.390)	0.388
ASA 3	0.532 (0.037 - 7.636)	0.642
ASA 4	N/A	N/A
Radiotherapy (RTX)		
No RTX	Reference	
Pre-Reconstruction RTX	0.607 (0.142 - 2.594)	0.500
Post-Reconstruction RTX	0.522 (0.143 - 0.979)	0.346
Perforator Diameter (mm)	0.510 (0.176 - 1.482)	0.216
Shortest Distance Between Perforator and Flap Edge (mm)	0.969 (0.940 - 0.991)	<0.001*
Ischaemia Time (min)	1.05 (0.932 - 1.107)	0.184

Multivariate logistic regression model displaying predictive factors for perfusion-related complications (PRCs). Significance (p<0.05) was indicated with an asterisk (*)

### Shortest Distance Between the Perforator and Flap Edge as a Predictor for Perfusion-Related Complications

**Figure 1:**
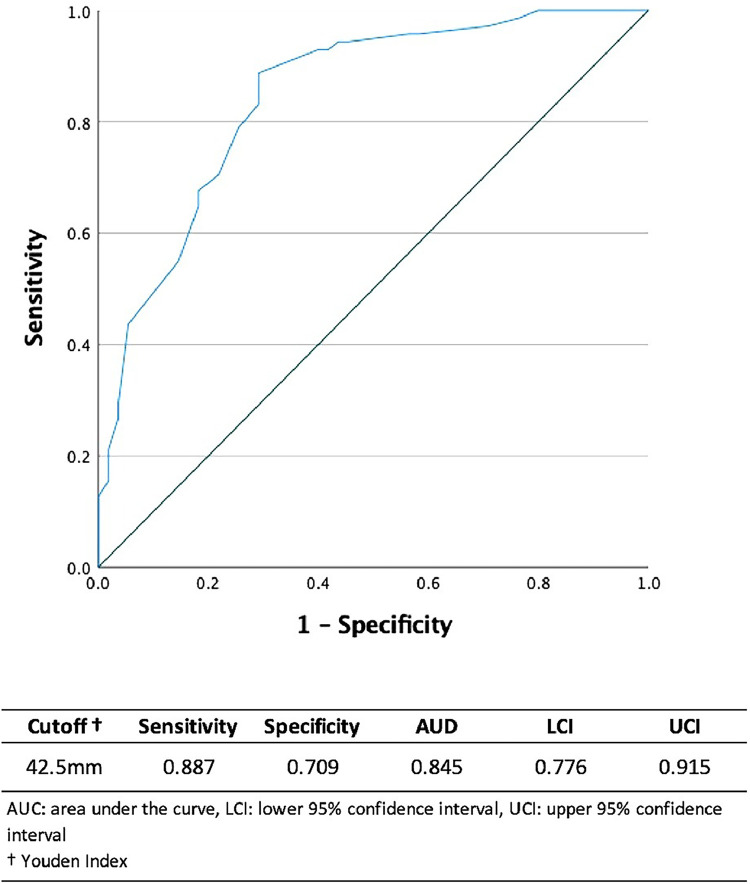
ROC Curve Analysis for the Relationship Between ‘Shortest Distance Between Perforator and Flap Edge’ and ‘Perfusion-Related Complications’

After reaching significance in the regression model, an ROC curve was analysed to identify the cut-off point for the shortest distance between the perforator and flap edge for distinguishing the probability of PRC development. This value was identified to be 42.5 mm, and area under the curve was 0.845 (sensitivity 0.887, specificity 0.709, 95% CI 0.776 - 0.915) (Figure 1).

## Discussion

Success of a DIEP flap breast reconstruction hinges on adequate perfusion throughout the flap. Several perforator characteristics, such as perforator centrality, diameter, row and number of perforators, are thought to influence perfusion. However, consensus on the significance and hierarchy of each perforator-related factor remains elusive. This study aimed to investigate the impact of several perforator-related characteristics on the development of PRCs in DIEP flap breast reconstruction.

The main finding of this study was the significant association between perforator centrality (represented by the shortest distance between the perforator and flap edge) and PRC development. The results showed that as the distance between the perforator and flap edge increased, there was a lower chance of developing PRCs. Although this has long been suspected by breast microsurgeons, this study represents the first scientific demonstration that in DIEPs, PRC rate increases as the distance between the perforator and flap edge decreases. Our results are supported by a recent study which showed that DIEPs based on perforators located in the horizontal middle third experienced significantly fewer PRCs compared with DIEPs based on perforators located in the horizontal superior or inferior thirds.^22^ Findings similar to that in our study have also been reported in anterolateral thigh flaps.^23^

The clinical implications of our results are relevant for the planning and execution of a DIEP flap breast reconstruction. Breast microsurgeons consider various perforator-related characteristics, such as perforator diameter, number of perforators, perforator row, and relative location of the perforator within the flap, to maximise flap perfusion. However, optimising perforator selection requires compromise between the various characteristics, such as choosing a larger more peripherally located perforator or a smaller more centrally located perforator. The findings of this study can aid surgeons in such decision-making processes, as our results suggest that perforator centrality exerts a greater impact on PRC development than perforator diameter. However, in practice, these decisions are often more complex. For example, in some cases, it may be desirable to shift the flap design upwards to facilitate centrality of a large perforator, at the expense of a higher donor-site scar. In cases where this scenario is not acceptable to the patient, additional perforators may be incorporated to enhance perfusion. These scenarios aim to highlight the nuanced nature of such decisions. Hence, through ROC analysis, we also calculated an indicative cut-off value for the shortest distance between the perforator and flap edge, which was 42.5 mm. This approach intends to simplify and facilitate the adoption of our primary finding into clinical practice.

Various imaging techniques can be applied pre- and intraoperatively to help the surgeon make decisions regarding perfusion of the DIEP flap. Preoperatively, CTA has become the mainstay imaging technique to aid in perforator selection, due to its widespread availability and high accuracy in determining the calibre, position and intramuscular course of abdominal perforators.^24^^,^^25^ CTA also can reveal the three-dimensional structure of the perforator branches in the subcutaneous tissue. Colour Doppler ultrasonography (CDU) can also be used for preoperative perforator mapping. Recent studies show that CDU can provide greater accuracy in localising perforators compared with CTA.^26^^,^^27^ These results, combined with the advantage of avoiding radiation exposure associated with CTA, may increase the popularity of CDU for preoperative perforator mapping. However, more studies are needed to support these results. Indocyanine green angiography (ICG-A) is another imaging modality used to assess and optimise perfusion in DIEP flaps. ICG-A has gained significant popularity in the recent years for its application in DIEP flap breast reconstruction owing to its high sensitivity in detecting perfused tissue in real time.^28^ Once injected intravenously, ICG binds to the endovascular plasma proteins and emits fluorescence on binding, which can be detected using a receiver.^29^^,^^30^ This allows the surgeon to intraoperatively visualise sufficiently perfused tissue and can resect inadequately perfused parts of the flap, thereby reducing the incidence of PRCs.^31^ However, the potential for ICG-A to improve patient outcomes in breast microsurgery is currently limited by its lack of widespread availability.

Although this study specifically examined single perforator DIEP flaps, it is important to acknowledge the ongoing debate regarding whether a single perforator or multiple perforators offer superior perfusion. Several studies support the preferential use of multiple perforator-based flaps and show that flaps based on multiple perforators experience lower rates of fat necrosis compared with flaps based on a single perforator.^1^^,^^18^^,^^32^ A common explanation for these findings is that flaps with more perforators have a more distributed and robust perfusion. Conversely, several studies showed no difference in the PRC rates among flaps based on single versus multiple perforators.^19^^,^^33^^,^^34^ However, other studies indicate single perforator-based flaps to be the superior choice,^35^^,^^36^^,^^37^ for various reasons. One study supported this proposition by demonstrating that mean arterial flow rate is higher in single perforator-based DIEP flaps,^37^ whereas another study showed that single perforator-based flaps possess a greater total pedicle flow and greater perfusion of zone 4,^36^ indicating a more globally perfused flap. Among the studies supporting the use of single perforator-based flaps, one made the clear distinction that when comparing single versus multiple perforator-based flaps, they were exclusively using flaps based on the single-dominant perforator for the single perforator group.^35^ In this study, the rate of PRCs did not vary between the 2 groups. However, it is not evident what the average diameter of the single-dominant perforator was in the study. They posit that single perforator-based flaps can be safely used if based on the largest perforator. If there is no single largest perforator, 2 perforators can be used if they arise from the same row; otherwise, the additional dissection across the intervening muscles nullifies the benefit of a perforator-based flap, and conversion to a muscle-sparing TRAM may be appropriate. Considering the significant heterogeneity in the literature regarding the choice between single versus multiple perforator-based DIEP flaps, further studies are needed to establish optimal perfusion strategies.

Furthermore, selection of perforator row in DIEP flap perfusion remains a commonly discussed subject, marked by the conflicting evidence in the literature. Previous anatomic studies by Wong et al. showed that medial row perforators preferentially supply Hartrampf zone 2 than zone 3, while lateral row perforators preferentially supply Hartrampf zone 3 than zone 2.^38^ This study also suggests that medial row perforators supply a greater vascular area compared with lateral row perforators. However, contradictory results found by a recent systematic review showed a heightened risk of fat necrosis in DIEP flaps relying on medial row perforators compared to their lateral counterparts.^39^ Although, our investigation aligns with existing studies indicating that perforator row might not exert a significant influence on the development of PRCs.^19^^,^^34^ Decisions regarding deep inferior epigastric artery branch harvest should be based on the clinical and radiological assessment of perforator quality, rather than the theoretical benefit of medial branch perforators.

Although perforator centrality was the only factor that maintained significance in multivariate analysis, perforator diameter was notably significant in univariate analysis. We only identified one other study that examined the influence of DIEP flap perforator diameter on PRCs. The results of this study indicated that the perforator diameter did not exert a significant impact on the extent of insufficiently perfused tissue within the flap.^34^ More studies are needed to ascertain the influence of this parameter on the perfusion of the DIEP flap.

This study has the following limitations. First, although the sample size can be considered large, as 292 patients were included, 65 cases of PRCs is relatively small; hence, significant relationships between the potential risk factors and PRCs may be hidden. Furthermore, as only 5 variables were included in the multivariate model, predictors related to PRCs may have been confounded by variables not included in the model. Moreover, we used the shortest distance between the perforator and flap edge to denote perforator centrality. This absolute measurement does not account for the variations in flap sizes between individuals, as the same distance between the perforator and flap edge would be proportionally different in a small versus large flap. Moreover, the presence of fat necrosis was logged into the prospective database based on clinical judgement, which can contribute to false positive or false negative cases of fat necrosis. Additionally, this study only evaluated the impact of the arterial component of the perforator. Although the venous component also exerts an influence on the development of PRCs, this aspect was not evaluated in this study. In addition, missing data for some investigated variables reduced the statistical power and increased the risk of selection bias. Furthermore, because measurement of the shortest distance between perforator and flap edge in this study was conducted intraoperatively using a simple surgical ruler, fine interpretation of the distance could be prone to human error. Finally, as this study included only patients under the care of a single surgeon, from a single centre, the results identified may not be widely generalisable.

## Conclusions

Currently, there is no consensus regarding which perforator characteristics significantly impact the rates of PRCs in DIEP flap breast reconstruction. This study showed that the shortest distance between the perforator and flap edge is a strong predictor for the development of PRCs in DIEP flap breast reconstruction. As there is usually a balance of several factors when planning and performing DIEP flap breast reconstruction, the findings of this study can help guide surgeons in clinical decision making. Owing to the variation in the literature regarding which perforator characteristics significantly impact DIEP flap perfusion, further studies are needed in this area to investigate the impact of perforator number and perforator diameter on the rates of PRCs. More studies are needed to investigate the impact of perforator centrality to support the results of this study.

## Conflict of interest statement

None.
