# Corrigendum to “Perforator-related risk factors for perfusion-related complications in DIEP flap breast reconstruction” [JPRAS Open 44 (2025) 390–401]

**DOI:** 10.1016/j.jpra.2025.07.002

**Published:** 2025-07-10

**Authors:** Dernas Suhail, Ryan Faderani, Afshin Mosahebi, Augustine Akali

**Affiliations:** 1 Department of Plastic Surgery, Morriston Hospital, Swansea SA6 6NL, UK; 2 Department of Surgery and Interventional Sciences, University College London, London NW3 2PS, UK; 3 Department of Plastic Surgery, Royal Free Hospitals NHS Trust, London NW3 2QG, UK; 4 Department of Plastic Surgery, Hull University Teaching Hospitals NHS Trust, Hull HU16 5JQ, UK

The authors regret that the author list of our article entitled “Perforator related risk factors for perfusion related complications in DIEP flap breast reconstruction” was submitted and subsequently published with the authors’ initials alongside the full names.

The consequence of this is that the article is not searchable under our names on bibliographic databases (eg, Pubmed, EMBASE, etc.). Hence, we would immensely appreciate if the author list could be changed from:

— Current: Dernas Suhail Ds, Ryan Faderani Rf, Afshin Mosahebi Am, Augustine Akali Aa
— Requested change: Dernas Suhail, Ryan Faderani, Afshin Mosahebi, Augustine Akali

Thank you very much and we apologize for the inconvenience caused.

